# Emerging pneumococcal serotypes in Iraq: scope for improved vaccine development

**DOI:** 10.12688/f1000research.132781.2

**Published:** 2023-12-28

**Authors:** Haider N. Dawood, Ali H. Al-Jumaili, Ahmed H. Radhi, Delan Ikram, Ali Al-Jabban

**Affiliations:** 1 Al Immamin al kadhimin Medical City, Baghdad, 10011, Iraq; 2 Merit consultant pediatrician, Baghdad, 10011, Iraq; 3 F.i.c.m.s/ C.M, Center for disease control and prevention, Baghdad, Iraq; 4 Pfizer, Baghdad, Iraq

**Keywords:** Iraq, Pneumococcal disease, Pneumococcal serotypes, Pneumococcal vaccines, Serotype replacement

## Abstract

Pneumococcal disease is a global public health concern as it affects the young, aged and the immunocompromised. The development of pneumococcal vaccines and their incorporation in the immunization programs has helped to reduce the global burden of disease. However, serotype replacement and the emergence of non-vaccine serotypes as well as the persistence of a few vaccine serotypes underscores the need for development of new and effective vaccines against such pneumococcal serotypes. In the Middle East, places of religious mass gatherings are a hotspot for disease transmission in addition to the global risk factors. Therefore, the periodic surveillance of pneumococcal serotypes circulating in the region to determine the effectiveness of existing prevention strategies and develop improved vaccines is warranted. Currently, there is a lack of serotype prevalence data for Iraq due to inadequate surveillance in the region. Thus, this review aims to determine the pneumococcal serotypes circulating in Iraq which may help in the development and introduction of improved pneumococcal vaccines in the country.

## Introduction

*Streptococcus pneumoniae*, the causative agent of pneumococcal disease, is responsible for devastating morbidity and mortality among children <5 years of age, adolescents, adults >60 years of age, the immunocompromised population, patients with chronic diseases, and smokers. ^1^^,^^2^ It is a commensal microbe that normally colonizes the upper respiratory mucosa of humans, which in turn facilitates its transmission. ^3^ However, several bacterial and host factors promote pathogen invasion into sterile body sites, thereby causing severe invasive pneumococcal disease (IPD) manifestations, such as septicemia, meningitis, and bacteremic pneumonia. ^3^^,^^4^ As a public health prevention strategy, the World Health Organization (WHO) has recommended the global inclusion of pneumococcal conjugate vaccines (PCVs) into routine infant immunization programs. ^5^ Currently, 148 countries have adopted this strategy, which has significantly reduced the burden of pneumococcal disease among children (<5 years) from 14.5 million episodes and 826,000 deaths in the pre-PCV period (2000) to 9.18 million cases and 318,000 deaths in 2015. ^6^^–^^8^ In addition, the pneumococcal polysaccharide vaccine (PPSV) was developed to prevent IPD among the elderly (≥65 years) and high-risk populations (≥2 years). ^9^ However, an increase in the emergence of nonvaccine serotypes (NVTs) or serotype replacement has been observed due to the vaccine’s selection pressure. ^10^^–^^13^

Due to the lack of adequate surveillance in the region, limited serotype prevalence data are available for Iraq. This review aims to revise and shed light on the *S. pneumoniae* serotypes circulating in the country. All systematic reviews used in this manuscript were found to be reliable in terms of heterogeneity and analysis. We hope that this review will aid readers (researchers and policymakers) in making informed decisions regarding the development and introduction of improved pneumococcal vaccines.

## *S. pneumoniae* serotypes

Pneumococci possess several virulence factors, of which the polysaccharide capsule is the most important, as it is an essential armor against phagocytosis and aids colonization by overcoming its mucus-mediated clearance. ^14^^,^^15^ The capsule is also the target antigen for the development of multivalent vaccines against this pathogen. Pneumococcal serotypes are differentiated based on antigenic differences and the chemical composition of these capsular polysaccharides. ^14^ A serogroup includes serotypes that have common serological properties ( *i.e.*, cross-reactive antibodies). ^16^

While most pneumococcal capsules are generally anionic, the capsule of serotype 1 exists as a zwitterion and those of serotypes 7A, 7F, 14, 33A, 33F, and 37 have been reported to be uncharged. ^16^ The capsule of serotype 14 is less soluble than other pneumococcal polysaccharide capsules. ^16^ The capsule strands are normally linked to the bacterial surface *via* covalent linkages to the peptidoglycan, except in the case of serotype 3, where noncovalent interactions with phosphatidylglycerol have been reported. ^17^ It has been suggested that the release of the capsular polysaccharide of serotype 3 strains reduces antibody-mediated protection and is responsible for the reduced efficacy of the currently available PCV against serotype 3. ^18^

Serotype distribution varies temporally and geographically and is also influenced by age, the presence of antimicrobial resistance genes, as well as disease syndrome and severity. ^5^ The establishment of this commensal nasopharyngeal flora occurs within the first year of birth. ^19^ Nasopharyngeal carriage determines disease development as well as pathogen dissemination. Infants and young children have higher carriage rates (27–85%) than adults (~10%). ^5^^,^^14^ It has been reported that a carriage rate of 30–40% is maintained till the age of 9 years, progressively declining thereafter. ^14^ In fact, the low carriage rates among adults indicates immunological protection upon previous exposure. ^14^^,^^19^ Variations in carriage rates have been noted to depend upon the local epidemiology, with higher rates noted in socioeconomically weaker countries and in impoverished communities with low vaccination rates. ^5^^,^^14^

Currently, more than 100 serotypes of *S. pneumoniae* are known, of which only about 23 cause 80–90% of invasive disease where the pathogen migrates to sterile sites within the body ( Table 1). ^1^^,^^20^^–^^22^ Young children (<5 years) and adolescents/adults are colonized by different pneumococcal serotypes. ^14^ Between 2004 and 2009, the most prevalent global serotypes among children <5 years were reported to be 19A, 19F, 14, and 6A, while those most commonly circulating among adults (>16 years) were serotypes 19A, 3, 6A, and 7F. ^23^ Additionally, a few serotypes show a propensity towards specific organ systems. Serotypes 6, 10, and 23 are frequently isolated from the meninges during meningitis, while serotypes 1 and 3 favor lung colonization during pneumonia. ^22^

**Table 1.** Colonization or invasive potential of *S. pneumoniae* serotypes.

Study parameters	Johnson *et al.*, 2010 ^21^	Brueggemann *et al.*, 2003 ^24^	Sleeman *et al.* 2006 ^25^	Kronenberg *et al.*, 2006 ^26^	Sa-Leao *et al.*, 2011 ^27^	Rivera-Olivero *et al.*, 2011 ^28^	Shouval *et al.*, 2006 ^29^	Yildirim *et al.*, 2010 ^30^	Balsells *et al.*, 2018 ^51^
Study period	1980−2007	1994−2001	1995−1997	2001−2004	2001−2003	2006−2008	2000−2004	2003−2009	Year when PCV was available−2015
Country	Global	UK	UK	Switzerland	Portugal	Venezuela	Israel	USA	Global
Predominantly invasive serotypes	1, 5, 6A, 6B, 14, 19F, 23F	1, 4, 7F, 9V, 14, 18C, 19A	1, 4, 5, 7F, 8, 9A, 9V, 12F, 14, 18C, 19A	1, 4, 5, 7F, 8, 9V, 14, 19A	1, 3, 4, 5, 7F, 8, 9N, 9L, 12B, 14, 18C, 20	1, 3, 5, 7F, 14, 18, 19F	1, 5, 12F, 9V, 18C, 19A, 19F	3, 7F, 18C, 19A, 22F, 33F	1, 7F, 12F, 19A, 8, 24F, 33F, 6C, 15A, 15BC, 16F, 23B
Predominantly colonizing serotypes	-	3, 6A, 6B, 8, 9N, 15B/C, 19F, 21, 23F, 38, 33F	3, 6A, 6B, 9N, 10A, 11A, 16F, 15B/C, 19F, 20, 21, 22F, 23A, 23F, 33F, 35F, 38	3, 6A, 6B, 7, 10, 11, 15, 19F, 23, 23F	6A, 6B, 11A, 15B/C, 16F, 19F, 23F, 34, 35F, 37	6A, 6B, 19A, 23F	3, 6A, 6B, 11A, 14, 15A, 15B/C, 21, 23F, 35B	11A, 15A, 15B/C, 19F, 35F, 6C, 23A, 35B	-

PCV, pneumococcal conjugate vaccine; UK, United Kingdom; USA, United States of America.

The capsular serotype determines the duration of nasopharyngeal carriage, as well as its invasive potential. ^14^^,^^24^^,^^25^ Serotypes with poor immunogenicity often colonize for longer durations. ^14^^,^^19^ In most published reports, serotypes 1, 4, 5, 7F, 8, 12F, 14, 18C, and 19A showed high invasive potential, while 6A, 6B, 11A, 15B/C, and 23F were less invasive and showed a higher colonization frequency. ^14^^,^^24^^–^^31^ The serotypes causing acute otitis media in children (<18 years) globally were reported to be 3, 6A, 6B, 9V, 14, 19A, 19F, and 23F. ^32^ Among adults, serotypes 1 and 19A predominantly caused invasive pneumococcal pneumonia, and serotype 14 was responsible for nonbacteremic pneumonia incidents during the post-PCV (PCV7) introduction phase. ^33^^,^^34^ For bacteremic pneumonia, serotypes 1, 7F, and 8 were associated with a lower risk of death, while infections with serotypes 3, 6A, 6B, 9N, and 19F resulted in increased mortality. ^35^ Serotype 1 is one of the most frequently isolated pathogen during IPD incidents, but rarely colonizes the nasopharynx. ^36^ It is mostly recovered from young adults without any comorbidities and has the potential to cause disease outbreaks and epidemics. ^36^ Among pediatric patients, serotype 1 was often reported to be associated with empyema complications post-pneumococcal infection in the period prior to PCV use, while serotypes 3, 7F, 14, and 19A have emerged post-PCV7 introduction. ^14^^,^^37^^–^^41^ Sirotnak *et al*. reported that *S. pneumoniae* serotype 1 also induced peritonitis among female children in the UK. ^42^ The mucoid serotype 3 has a thicker capsule, greater virulence, and higher mortality rate than other strains and is the second most common isolate in adult IPD cases. ^43^^–^^45^ It is associated with severe clinical manifestations, such as empyema, cardiotoxicity, bacteremia and meningitis, with a fatality rate of 30–47%. ^17^ Moreover, it resists antibody-mediated clearance, as the antibody titers required to confer protection are not elicited by the current conjugate vaccine. ^17^^,^^18^ Prior to PCV introduction, serotype 14 was reported to be the most common cause of pneumococcal-associated hemolytic uremic syndrome among children, which shifted to serotypes 1, 3, 7F, and 19A in the post-PCV7 era, with serotype 3 being the most predominant. ^14^^,^^46^^–^^49^ Among adults, the following serotypes have been reported to be associated with an elevated risk of: empyema (serotypes 1, 3, 5, 7F, 8, 19A), necrotizing pneumonia (serotype 3), septic shock (serotypes 3, 19A), meningitis (serotypes 10A, 15B, 19F, 23F), reduced quality-adjusted life-years (serotypes 15B, 3, 10A, 9N, 19F, 11A, 31), and increased case-fatality rates (serotypes 3, 6B, 9N, 11A, 16F, 19F, 19A). ^50^ Among vaccinated children <5 years, serotypes 1, 7F, and 12F showed a higher invasive potential than serotype 19A. ^51^ Further, the NVTs 8, 12F, 24F, and 33F were at the upper end of the invasiveness spectrum in this cohort. ^51^

The serotypes 19A, 6A, 19F, 6B, 15A, 9V, and 14 have been reported to exhibit erythromycin resistance, while 19A, 19F, 35B, 6A, 6B, 23A, 9V, 15A, and 14 demonstrated penicillin resistance. ^23^ Serotype 19A strains have been reported to be multidrug resistant and are prevalent globally. ^52^^,^^53^

## Pneumococcal vaccines and serotype replacement

The first PCV offered protection against 7 pneumococcal serotypes (PCV7: 4, 6B, 9V, 14, 18C, 19F, 23F) and was licensed in 2000. ^54^ Currently, 26 countries use the 10-valent (PCV10: PCV7 + 1, 5, 7F) formulation and 114 countries use the 13-valent (PCV13: PCV10 + 3, 6A, 19A) formulation, while 7 countries use both. ^5^^,^^8^^,^^54^ In India, an alternate 10-valent formulation, Pneumosil ^®^ (Serum Institute of India), has been adopted. In the USA, two new polyvalent conjugate vaccines, PCV15 (PCV13 + 22F, 33F) and PCV20 (PCV15 + 8, 10A, 11A, 12F, 15B), are now available for use in adults ≥18 years. ^55^ Merck Sharp & Dohme’s (MSD’s) (PCV13 + 2, 8, 9N, 10A, 11A, 12F, 15B, 17F, 20, 22F, 33F) and Affinivax’s (PCV13 + 2, 8, 9N, 10A, 11A, 12F, 15B, 17F, 20B, 22F, 33F) 24-valent vaccine formulations are under clinical trials. ^56^ Additionally, a 23-valent pneumococcal polysaccharide vaccine (PPSV23: 1, 2, 3, 4, 5, 6B, 7F, 8, 9N, 9V, 10A, 11A, 12F, 14, 15B, 17F, 18C, 19A, 19F, 20, 22F, 23F, 33F) was licensed in 1983 for use in the elderly and high-risk populations. ^54^ Furthermore, Inventprise plans to develop a vaccine to target important emerging serotypes such as 2, 16F, 24F and 35B while Vaxcyte has also announced a 30-valent preclinical PCV to target newly emerging IPD strains and antibiotic resistance. ^56^ Table 2 lists the different pneumococcal vaccines and the serotypes they cover.

**Table 2.** Pneumococcal vaccines.

S. No.	Vaccine	Serotypes
1.	PPSV23 (Pneumovax ^®^ 23, MSD)	1, 2, 3, 4, 5, 6B, 7F, 8, 9N, 9V, 10A, 11A, 12F, 14, 15B, 17F, 18C, 19F, 19A, 20, 22F, 23F, 33F
2.	PCV7 (Prevnar ^®^, Pfizer )	4, 6B, 9V, 14, 18C, 19F, 23F
3.	PCV10 (Synflorix ^®^, GlaxoSmithKline)	1, 4, 5, 6B, 7F, 9V, 14, 18C, 19F, 23F
4.	PCV10 (Pneumosil ^®^, SII)	1, 5, 6A, 6B, 7F, 9V, 14, 19A, 19F, 23F
5.	PCV13 (Prevnar 13 ^®^, Pfizer)	1, 3, 4, 5, 6A, 6B, 7F, 9V, 14, 18C, 19A, 19F, 23F
6.	PCV15 (Vaxneuvance ^®^, MSD)	1, 3, 4, 5, 6A, 6B, 7F, 9V, 14, 18C, 19A, 19F, 22F, 23F, 33F
7.	PCV20 (Prevnar 20 ^®^, Pfizer)	1, 3, 4, 5, 6A, 6B, 7F,8, 9V,10A, 11A, 12F, 14, 15B, 18C, 19A, 19F, 22F, 23F, 33F

PPSV: Pneumococcal polysaccharide vaccine; PCV: Pneumococcal conjugate vaccine; MSD: Merck Sharp & Dohme; SII: Serum Institute of India.

The impact of PCVs in reducing pneumococcal colonization and invasive disease by vaccine serotypes in vaccinated children, as well as herd protection among the unvaccinated population, is well documented. ^57^^–^^60^ Although pneumococcal vaccines have had a tremendous impact on the global pneumococcal disease burden, serotype replacement ( *i.e.*, the replacement of the vaccine serotypes [VTs] with nonvaccine serotypes) has slightly dulled the overall benefits of immunization. ^11^

Alterations in the serotype prevalence among pneumococcal populations can originate from both serotype replacement as well as serotype (capsular) switching. ^61^ Serotype replacement refers to the expansion of NVTs within the population. ^62^^,^^63^ On the other hand, serotype switching, or capsular switching, is the change in a serotype from a single clone that occurs due to alterations in the *cps* locus which is responsible for capsular polysaccharide synthesis. ^61^ These 2 events are not mutually exclusive, as capsular switch variants often expand within the population. ^61^^,^^62^

## Global pneumococcal serotype prevalence

In the period preceding worldwide PCV introduction, the most commonly circulating pneumococcal serotypes in children (<5 years) were reported to be 1, 5, 6A, 6B, 14, 19F, and 23F, which accounted for 58–66% of global IPD cases. ^21^ During this period, PCV7-related serotypes were responsible for 49–82% of global childhood IPD episodes. ^21^ Upon PCV7 implementation, the percentage of IPD cases caused by PCV7 serotypes reduced to approximately 14.8% and to 12.5% after the introduction of the higher-valent PCVs ( *i.e.*, PCV10/13). ^64^^,^^65^ The PCV10-specific serotypes 1, 5, and 7F accounted for 16.3% of overall childhood IPD cases in regions where PCV7 had been introduced, while the figure was 9.2% in post-PCV10/13 implementation settings. ^64^ Following the introduction of PCV7 and PCV10/13, the most common PCV13 serotypes were 19A, 3, and 6A. ^45^^,^^64^ Serotype 19A was the most prevalent (21.8% cases) among pediatric IPD cases in the post-PCV7 period, and was responsible for 14.2% of incidents in regions with higher-valent vaccine introduction. ^64^ The global increase in the prevalence of serotype 19A in the PCV13 period is a cause of concern, as it indicates reduced vaccine efficacy against this serotype. ^45^ Furthermore, this serotype is also globally associated with high multidrug resistance (MDR) potential. Wantuch and Avci also highlight PCV13's reduced effectiveness against serotype 3 prevalence, which has remained nearly constant in the pre-(1999–2000) and post-PCV (2010–2011) periods. ^45^

Globally, about 29.4% and 42% of childhood IPD cases were caused by non-PCV13 serotypes in post-PCV7 and post-PCV10/13 administration settings, respectively. Serotype 22F was the most prevalent (5% of childhood IPD episodes) in regions where PCV10/13 had been introduced, followed by 12F, 33F, 24F, and 15C (~4% episodes each). Serotypes 15B, 23B, 10A, and 38 were responsible for 3.4–3.7% of cases globally. However, several variations in the prevalence of these serotypes were observed across different regions. In several regions, except in the EMR, serotypes 22F, 12F, and 33F were responsible for 4–16% of childhood IPD cases. Serotype 24F was prevalent in the Western Pacific region and Europe (but not North America). Serotype 38 was more prevalent in North America and 12F, 15C, and 10A in Europe. ^64^ In Japan, a nationwide surveillance study was conducted between 2012 and 2014 among pediatric (2 months–16 years) patients that revealed the emergence of IPD due to NVTs 24F and 15A post-approval of PCV13 use in routine vaccination. ^66^ Ubukata *et al*. found that the prevalence of 9 (10A, 12F, 15A, 15B, 15C, 22F, 24F, 33F, and 35B) and 5 serotypes (12F, 15C, 22F, 23A, and 35B) increased significantly among children and adults, respectively, during the PCV13 period. Notably, a rapid increase was observed in 15A and 35B. ^67^ The NVT 24F was recently reported to be a cause of concurrent bacteremia among 22-month-old twins who had been fully vaccinated with PCV13, with the last dose administered 6 months prior to hospitalization. ^68^ This serotype also showed a high invasive potential (pneumococcal meningitis) post-PCV13 implementation in France in the pediatric population along with serotype 12F. ^69^^,^^70^

## Pneumococcal serotype prevalence in the Eastern Mediterranean Region

The EMR has a substantial burden of lower respiratory tract infections, with pneumococci contributing maximally to the mortality rate (16.6 per 100,000). ^71^ In 2015, this region reported 37,100 pneumococcal deaths and 968,000 pneumococcal disease cases. ^7^ High-income countries within the region have introduced PCV into their National Immunization Programs (NIPs), and several low-income countries have received support from Gavi, the Vaccine Alliance, for the same. ^71^ Several middle-income countries have now introduced PCV, except for a few countries such as Iran, Jordan and Egypt. ^8^ Currently, a majority of EMR countries have incorporated PCV13 use, while Morocco, Pakistan, and Tunisia use the 10-valent Synflorix ^®^ vaccine. ^8^

The Gulf countries of the Middle East exhibit a high pneumococcal disease burden, with risk factors similar to those existing globally, such as age-related risks, chronic disease-related risks, as well as risks arising due to immunodeficiencies, smoking, and alcoholism. ^72^^,^^73^ Additionally, religious mass gatherings occurring within this region act as hotspots for pathogen dissemination among pilgrims (especially those with comorbidities) during Hajj, Umrah, and Arba’een. ^74^^–^^77^ However, inadequate surveillance as well as inconsistent reporting methods are associated with an underestimation of pneumococcal diseases burden in the Gulf countries. ^78^ Thus, the active surveillance of pneumococcal serotypes prevalent within this region would help determine the effectiveness of the adopted immunization strategies and aid in developing novel measures to control disease transmission and outbreaks. Currently, the EMR has a vaccination coverage of 52% for 3 doses of PCV. ^79^ Further, it has been reported that the predominant MDR pneumococcal strains in the Arab League belong to serotypes 19F, 23F, 6B, and 19A. ^80^^–^^82^ Table 3 provides an age-wise stratification of serotype prevalence in the EMR and correlation with antibiotic resistance (where available).

**Table 3.** Age-wise stratification and antibiotic resistance among *S. pneumoniae* isolates in the Eastern Mediterranean Region.

Study parameters	Study period	Study population	Serotype prevalence	Antibiotic resistance
***Iran***				
Houri *et al.*, 2017 ^84^	July 2013−March 2016	<5 years	23F, 19F, 19A, 9V	Penicillin-non-susceptible: 21% (resistance: 17%; and intermediate resistance: 4%)
Ghahfarokhi *et al.*, 2020 ^86^	February−September 2015; July 2018−March 2019	≥1 month	Overall: 23F, 14, 3, 19F, 19A, 6A, 6B, 9V, 18C ≤5 years: 19A, 3, 23F, 14 ≥64 years: 23F	Penicillin non-susceptible: 23F, 14, 19F, 3, 19A (period I); 23F, 19F, 3, 14, 19A, 6A, 6B (period II) MDR: 14, 23F, 19F, 3, 18C, 9V, 19A (period I); 19A, 23F, 19F, 3, 6A, 14 (period II)
Chamkaleh *et al.*, 2020 ^85^	January 2000−August 2019	All ages	Invasive: 23F, 19F, 19A, 6A/B, 9V, 11A Non-invasive: 6A/B, 19F, 14, 17F, 20	NR
***Iraq***				
Al-Sanouri *et al.*, 2021 ^89^	June 2018−May 2020	3 days to 91 years	NR	NR
Al-Saryi *et al.*, 2019 ^90^	NR	4 years	33C	NA *
***Kuwait***				
Mokaddas *et al.*, 2012 ^91^	August 2006−December 2011	All ages	≤5 years: 19F, 19A, 6A, 8, 15B (invasive); 19F, 23F (non-invasive) >50 years: 14, 3, 1, 19F, 8 (invasive); 19F, 23F, 6B, 14, 19A (non-invasive)	Penicillin resistant: 19A, 23F
Mokaddas and Albert, 2016 ^92^	Pre-vaccination: August 2003−July 2006; Post-vaccination: August 2006−July 2010 (period I); August 2010−July 2013 (period II)	All ages	<2 years: 14, 19F, 23F, 7F (pre-vaccination period); 6A, 19F, 18C, 6B, 5, 9V, 19A (post-vaccination period I); 19A, 23F (post-vaccination period II) 2−>65 years: 19F, 9V, 23F, 1, 14, 19A, 4, 6A, 5, 18C, 6B (pre-vaccination period); 3, 1,9V, 14, 19F, 5, 23F, 6A, 19A, 6B, 4, 18C (post-vaccination period I); 19A, 6A, 9V, 18C, 19F (post-vaccination period II)	Penicillin resistant: 6A, 6B, 8, 9L, 15B, 15C, 19A, 19F, 22A, 23F, 33D
Mokaddas *et al.*, 2018 ^78^	Pre-vaccination: August 2003−July 2006; Post-vaccination: August 2006−July 2010 (period I); August 2010−December 2016 (period II)	All ages	NR **	NR
***Lebanon***				
Hanna-Wakim *et al.*, 2012 ^94^	October 2005−December 2011	All ages	Overall: 19F, 6, 3, 14, 1, 19A <2 years:14, 19F, 6 >60 years: 3	Penicillin non-susceptible: 19F, 6, 14 MDR: 19F, 14
Reslan *et al.*, 2021 ^95^	2013−2019	All ages	24F	Macrolide and tetracycline resistant
***Morocco***				
Nzoyikorera *et al.*, 2021 ^98^	NR	Newborn (gestational age: 34 weeks)	17F ***	Tetracycline and chloramphenicol resistant
El Mdaghri *et al.*, 2012 ^82^	September 2007−August 2008	≤5 years	19F, 14, 23F, 6B, 19A, 1, 3, 5, 18C,	Penicillin non-susceptible: 62.5% Amoxicillin non-susceptible: 4.2% Erythromycin non-susceptible: 16.6% Trimethoprim–sulfamethoxazole non-susceptible: 33.3%
***Saudi Arabia***				
Shibl *et al.*, 2012 ^99^	2005-2010	≤5 years	23F, 19F, 6B, 5, 1	Penicillin resistant: 66% Erythromycin-resistant: 62%
Al-Sherikh *et al.*, 2014 ^101^	2009-2012	<15 years	23F, 6B, 19F, 18C, 4, 14, 19A	Penicillin resistant (36%): 23F, 6B, 19F, 18C, 4, 14 Co-trimoxazole resistant: 100% Erythromycin resistant: 77%
Alnimr and Farhat, 2017 ^102^	January 2012-December 2014	All ages	11A, 19A, 17F, 23F, 3, 19F	Penicillin and macrolide resistant: 67.9% (11A/D, 19A, 17F, 23F, 3, 19F, 14, 33F/A, 6A) Macrolide-monoresistant: 5.6% (1, 14, 33F/A) Penicillin resistant: 17% (11A/D, 19A, 17F, 14)
***Oman***				
Al-Jardani *et al.*, 2019 ^105^	June 2014-June 2016	≤5 years, 6-50 years and ≥51 years	Overall: 12, 15, 19F, 3,19A, 22 ≤5 years: 12, 19F, 23A, 16F ≥51 years: 3, 15, 19A, 19F, 22, 12, 11 6-50 years: 1, 12, 15,7F	56.8% of the isolates were non-susceptible to ≥1 antibiotic ****; penicillin-resistant: 40.9%; MDR: 18.9%

* Susceptible to penicillin, erythromycin, chloramphenicol, cefotaxime, and vancomycin.

** Number of isolates per year were reported for this study, serotypes were classified as PCV7, PCV13, PCV13 only, and non-PCV13.

*** Vertical transmission.

**** Penicillin, ceftriaxone, cefotaxime, meropenem, amoxicillin, oxacillin, erythromycin, clindamycin, chloramphenicol, trimethoprim/sulfamethoxazole, vancomycin, and levofloxacin.

MDR, multi-drug resistant; NA, not applicable; NR, not reported.

### Iran

Pneumococcal vaccines have not yet been included in the NIP of Iran and are only recommended for high-risk groups. Recently, a systematic review and meta-analysis of published reports between 2010 and 2017 showed that the overall prevalence of invasive *S. pneumoniae* infections is very low (2.5%) among Iranian children. ^83^ However, as the included studies covered only 3 geographical regions within the country, this study was not fully representative of the whole population and could not entirely estimate the overall *S. pneumoniae* prevalence. Among children (<5 years) in Tehran, serotype 23F was reported to be the most invasive circulating serotype, followed by 19F, 19A, and 9V. Serotype 19A was significantly associated with penicillin resistance. ^84^

Another systematic literature review on pneumococcal serotype distribution conducted among clinical and carrier Iranian patients between January 2000 and August 2019 revealed that 23F was the most commonly circulating serotype in Iran and was associated with IPD. Other serotypes that caused IPD included 19F, 19A, 6A/B, 9V, and 11A. Among carrier patients, 6A/B, 19F, 14, 17F, and 20 were the most frequent. ^85^

A study conducted between February 2015 and September 2015 and between July 2018 and March 2019 collected approximately 40 samples during each period from patients (1 month to 72 years of age) in Tehran. ^86^ About 38 of these samples were derived from invasive infections, of which 42% occurred in children ≤5 years. The pneumococcal serotypes in the samples were determined to be 23F, 14, 3, 19F, 19A, 6A, 6B, 9V, and 18C, of which the first 5 were the most common (in decreasing order). This is consistent with serotype prevalence data for other Asian countries. ^87^^,^^88^ The common serotypes isolated from invasive infections were 23F, 19A, and 14, while 3, 19F, and 23F were commonly associated with noninvasive disease. Among pediatric patients ≤5 years, the most prominent serotypes were 19A, 3, 23F, and 14, while 23F was predominant in adults ≥64 years. In the former age group, serotype 19A was observed in 35.2% of IPD cases, while serotypes 3, 23F, and 14 were predominantly non-IPD related. Overall, serotype 23F was frequently associated with penicillin resistance and was also predominant among MDR strains. A significant rise in serotype 19A MDR isolates was noticed among invasive infections in the second period of sample collection, possibly due to antibiotic selection pressure. ^86^

### Iraq

Iraq incorporated the use of PCV into the NIP in 2017. However, there are insufficient serotype surveillance data for Iraq. Most publications on *S. pneumoniae* in this region focus on its antimicrobial resistance, but do not identify the serotype of the isolate. Between June 2018 and May 2020, 41.6% of patients were confirmed to have pneumococcal meningitis in Iraq based on cerebrospinal fluid samples. ^89^ The age of patients ranged from 1 to 40 years, with a majority (83.7%) being under 5 years and 58.4% being less than a year old. The overall annual incidence rate (IR) of laboratory-confirmed pneumococcal meningitis in Iraq was 0.62/100,000, with a maximum IR of 1.56 in Karbala (site of Arba’een pilgrimage), 0.65 in Karkh, 0.58 in Al-Rusafa, 0.3 in Kirkuk, and a minimum of 0.09 in Maysan. However, as all Iraqi governorates were not covered in this study, the overall incidence rate for pneumococcal meningitis was likely underestimated. In a recent report, a nonvaccine *S. pneumoniae* serotype, 33C, was isolated from a hospitalized child with nephrotic syndrome and sepsis, which can be fatal. ^90^

### Kuwait

There are several publications that study the impact of PCV vaccinations, serotype prevalence, and pneumococcal drug resistance among the people of Kuwait. ^91^^–^^93^ The 7-valent PCV was introduced to the pediatric population in 2007 and was replaced with the higher-valent (PCV13) formulation in 2010. ^93^ In the period following PCV7's introduction (2006–2011), a majority (46%) of clinical pneumococcal isolates were derived from the adult population >50 years, where 27% of cases were found to be invasive. Although a lower percentage (23%) of isolates were obtained from pediatric (≤5 years) samples, IPD was responsible for nearly half (49%) of the cases in this age group. ^91^ The common serotypes circulating among children (≤5 years) during this period were 19F, 19A, 6A, 8, and 15B (invasive) and 19F and 23F (noninvasive). ^91^ Serotypes 14, 3, 1, 19F, and 8 were associated with invasive disease and 19F, 23F, 6B, 14, and 19A with noninvasive events among the adult population >50 years. ^91^ In comparison to the pre-PCV (10.33 isolates/year) and post-PCV7 (7.75 isolates/year) periods, PCV7-related serotypes showed a greater decline after PCV13 introduction, falling to 1.4 isolates/year. ^78^ An increased incidence of cases due to non-PCV7 serotypes 1, 6A, and 3 (which are included in PCV13) was reported post-PCV7 vaccine introduction. ^91^ After the introduction of the 13-valent vaccine, the 6 additional serotypes included in PCV13 showed a reduced frequency of occurrence (3.12 isolates/year) as compared to the pre-PCV (4 isolates/year) and post-PCV7 (7.5 isolates/year) periods, while the nonvaccine serotypes increased (13.25 isolates/year post-PCV7 and 11.52 isolates/year post-PCV13 introduction as compared to 6.33 isolates/year in the pre-PCV phase). ^78^

### Lebanon

In Lebanon, the private sector introduced PCV7 in 2006, followed by PCV10 and PCV13 in 2010. The universal introduction of the vaccine by the government was done in 2015. The Lebanese Inter-Hospital Pneumococcal Surveillance Program was a 6-year (October 2005–December 2011) program wherein 257 samples were isolated from patients with IPD affected mostly by pneumonia (46.5%), bacteremia (21.5%), and meningitis (17.2%). ^94^ The case-fatality rate was estimated to be 13.4%. Among affected patients, 33.1% were >60 years and 24.1% under 2 years. About 17.4% were penicillin resistant, with serotype 19F being the most common, followed by 6 and 14; 10.9% were MDR strains (19F and 14). IPD caused by vaccine serotypes was 41.4% for PCV7, 53.9% for PCV10, and 67.2% for PCV13. The most prevalent serotypes, overall, were those covered by PCVs: 19F, 6, 3, 14, 1, and 19A. In children <2 years, the most common serotypes/groups were 14, 19F, and 6. Serotype 3 was, surprisingly, the highest in those aged >60 years. The NVTs (not part of PCV7, 10, or 13) isolated were 22F, 33F, 11 A/D, 9N, 10A, 12F, 8, 15A, 15 B/C, 16F, 23A, 29A, 35B, and 38.

The NVT 24F has been reported to be an emerging serotype among patients with IPD (mostly [87.5%] <6 years of age with unknown pneumococcal vaccination status) in Lebanon, with 4 cases noted in 2019. ^95^ The genome sequencing of this serotype, isolated from samples collected between 2013 and 2019, showed that it is highly virulent and antimicrobial resistant. ^95^ The prevalence of this NVT among IPD cases in children has also been reported in the European and Western Pacific regions. ^64^^,^^68^^,^^70^^,^^96^^,^^97^

### Morocco

During a surveillance study conducted between September 2007 and August 2008 in Casablanca, serotypes 19F, 14, 23F, 6B, and 19A were found to be prevalent among pediatric (<5 years) IPD patients. ^82^ Recently, a report was published on early neonatal respiratory distress, revealing meningitis caused by serotype 17F *via* vertical transmission. ^98^

### Saudi Arabia

PCV7 was incorporated into the Saudi Arabian NIP in 2008 and was replaced with the 13-valent vaccine in 2010. ^99^ A multicenter, prospective study conducted between June 2007 and January 2009 estimated the incidence of confirmed IPD cases to be 2.5−21.6 per 100,000 children <5 years. ^100^ Between 2005 and 2010, serotypes 23F, 19F, 6B, 5, and 1 were commonly associated with invasive episodes among children <5 years. ^99^ This period showed a notable decline in the PCV7 serotype 18C and a significant rise in the PCV13 serotype 19A. ^99^ About 66% and 62% of isolates were reported to be penicillin and erythromycin resistant, respectively. ^99^ Similarly, between 2009 and 2012, the serotypes 23F, 6B, 19F, 18C, 4, 14, and 19A (in decreasing order) were reported to cause IPD among patients <15 years of age. ^101^ In this study, all pneumococcal isolates were found to be resistant to cotrimoxazole, while 77% and 36% were observed to be erythromycin and penicillin resistant, respectively. Penicillin resistance was higher among serotypes 23F, 6B, and 19F. ^101^ In the post-PCV13 introduction phase (January 2012 to December 2014), serotypes commonly isolated from the Eastern province of Saudi Arabia included 11A, 19A, 17F, 23F, 3 and 19F. ^102^ The previously rare but most prevalent serotype in this study, 11A (part of PPSV23), exhibited maximum penicillin resistance. Overall, 67.9% of isolates were resistant to both penicillin and macrolides, 17% to only penicillin and 5.6% to only macrolides. ^102^ These reports indicate that there is widespread drug resistance among pneumococcal isolates in Saudi Arabia. Of the pneumococcal isolates collected from 24 Saudi Arabian hospitals between January and December 2009, 33% were resistant to penicillin G, 26% to erythromycin, and 11% to ceftriaxone. ^103^

### Oman

In Oman, PCV7 was introduced in 2008, followed by the implementation of PCV10 in 2010, and PCV13 in 2012, in response to global reports on the emerging MDR serotype 19A. Among adults, PPSV23 use was implemented for high-risk patients. ^104^ IPD afflicted 45.5% of adult patients (≥51 years) in the period following PCV13 incorporation (2014–2016), with a maximum case-fatality rate of 21.7%. ^105^ In comparison, 26.5% of the affected population was ≤5 years, where the case-fatality rate was 14.2%. ^105^ Of note, 28% of the population was between 6 and 50 years, with a mortality rate of 8.1%. The major clinical presentations of IPD were pneumonia (52.3%), meningitis and septicemia (17.4% each). The most prevalent serotypes among all age groups were reported to be 12, 15, 19F, 3, 19A, and 22, while serotypes 1, 7F, 17F, 18C, and 9N/L had moderate prevalence. Serotypes 12, 19F, 23A, and 16F were commonly isolated from pediatric patients ≤5 years, whereas serotypes 3, 15, 19A, 19F, 22, 12, and 11 were common in adults (≥51 years). The most common serotypes in the 6–50 years’ age bracket were 1, 12, 15, and 7F. About 40.9% of these isolates were found to be resistant to penicillin.

## Conclusions

The reduction in the occurrence of vaccine-type serotypes since the introduction of PCVs points towards the might of vaccines in combating deadly infectious diseases. However, growing antibiotic resistance, serotype switching and replacement, and the persistence of a few vaccine serotypes (especially serotypes 3 and 19A) indicates that both vaccine-type and nonvaccine-type pneumococcal serotypes still remain a global public health concern. Thus, there is an ever-increasing need to combat IPD *via* effective, new-generation vaccines that utilize effective immune mechanisms, especially in the case of serotype 3, which can evade the immune system. Limited serotype prevalence data are available for Iraq due to lack of facilities for laboratory studies. In addition, issues such as vaccine inaccessibility in the public sector, war and conflict situation, displacement camps and climate change characterized by high temperature, dust storms, drought and increased desertification are additional challenges for a successful pneumococcal vaccination program in the country. Pneumococcal serotypes in Iraq are likely to be similar to those circulating in the neighboring countries due to similarities including ecological conditions and cultural practices like mass gatherings during worship. However, serotype replacement and capsule switching are regulated by selection pressure unique to the population. Thus, in Iraq and its neighboring countries in the EMR, improving surveillance would help provide the essential disease burden data required for refining vaccination strategies and improving outcomes. The active surveillance of NVT 33C, recently isolated from Iraqi children with nephrotic syndrome and sepsis, is essential to understand the degree of spread of the pathogen among Iraqi communities. In the PCV13 era, the emergence of serotype 24F in many regions of the world as one with maximum invasive potential and multidrug resistance warrants its periodic surveillance, as well as its inclusion in the next generation of pneumococcal vaccines. Improved polyvalency of vaccines, such as those under development by Inventprise and Vaxcyte, would help combat nonvaccine serotypes. Such an expanded coverage by the newer generation of vaccines is theoretically expected to reduce IPD cases caused by emerging non-PCV13 serotypes. In conjunction, continuous studies on molecular epidemiology of the pathogen within the EMR region would also help monitor antibiotic resistance patterns. We hope that this review guides policymakers and researchers to make informed decisions pertaining to the development and introduction of improved pneumococcal vaccines in Iraq.

## Data Availability

No data are associated with this article.
