# Supplementary material for: Emerging pneumococcal serotypes in Iraq: scope for improved vaccine development
Source: F1000Res. 2023 Dec 28;12:435. Originally published 2023 Apr 24. [Version 2] doi: 10.12688/f1000research.132781.2 (PMC10811421; doi:10.12688/f1000research.132781.2)
Supplement: Peer review history

# Reviewer report: Reviewer response for version 2

**DOI:** 10.5256/f1000research.160249.r233970

**Published:** 2024-01-25

**Authors:** Karthik Subramanian

**Affiliations:** 1 Rajiv Gandhi Center for Biotechnology, Thiruvananthapuram, Kerala, India

Dear Editor, I have no further comments to make.

Is the review written in accessible language?

Yes

Are all factual statements correct and adequately supported by citations?

Yes

Are the conclusions drawn appropriate in the context of the current research literature?

Yes

Is the topic of the review discussed comprehensively in the context of the current literature?

Yes

Reviewer Expertise:

Pneumococcal infections, host-pathogen interactions, bacterial pathogenesis

I confirm that I have read this submission and believe that I have an appropriate level of expertise to confirm that it is of an acceptable scientific standard.

# Reviewer report: Reviewer response for version 1

**DOI:** 10.5256/f1000research.145730.r208951

**Published:** 2023-10-16

**Authors:** Karthik Subramanian

**Affiliations:** 1 Rajiv Gandhi Center for Biotechnology, Thiruvananthapuram, Kerala, India

This review aims to summarize the emerging pneumococcal serotypes in Iraq and the middle eastern region to determine the effectiveness of the vaccines and help policy makers in Iraq to make informed decisions regarding introduction of improved vaccines. I believe this review is important and timely not just for the eastern Mediterranean region, but also has global relevance since it is a place of mass religious gatherings and pneumococcus is widely known for its horizontal gene transfer and global spread of resistant clones. In my opinion the authors can summarize the data and present it in a way that makes it easily comprehensible for readers rather than simply presenting the data on serotype prevalence. Below are my specific comments.

1. Most of the data presented in this review is drawn from systematic reviews. Since the inferences drawn in the review rely on these systematic reviews, it is important to assess the QC of these reviews such as clinical heterogeneity, statistical heterogeneity as well as models used for meta analysis. In some cases, the authors refer to systematic reviews from very few geographical regions of a country - e.g. Iran. How reliable are these studies? The reliability of the data presented is heavily dependent on the QC of these meta analyses. Therefore the authors need to ensure that they only present data from robust systematic reviews.
2. Section on pneumococcal serotypes - I suggest that the authors tabulate the pneumococcal serotypes associated with colonization vs. invasive disease along with the supporting references. 
3. Many times the authors list some serotypes as frequently associated with carriage and IPD. This does not make sense. 
  For e.g. page 6, under data from Iran, 19F is classified into both IPD and carrier populations. 
  Similarly, serotype 23F is listed under both carriage and IPD.
4. For the data on countries from EMR region, age-wise stratification of serotype prevalence and correlation with antibiotic resistance profile is needed.
5. The authors need to refrain from claiming that they can draw inference for Iraq from neighboring countries since although regions are located geographically closer, serotype replacement and capsule switching are regulated by selection pressure unique to the population. Even in this review there are unique serotypes associated with Iraq such as NVT 33C that is not found elsewhere in the region. 

Is the review written in accessible language?

Yes

Are all factual statements correct and adequately supported by citations?

Yes

Are the conclusions drawn appropriate in the context of the current research literature?

Yes

Is the topic of the review discussed comprehensively in the context of the current literature?

Yes

Reviewer Expertise:

Pneumococcal infections, host-pathogen interactions, bacterial pathogenesis

I confirm that I have read this submission and believe that I have an appropriate level of expertise to confirm that it is of an acceptable scientific standard, however I have significant reservations, as outlined above.

# Response

**Published:** 2023-12-19

**Authors:** Delan Ikram

**Affiliations:** Pfizer, Iraq

1. Most of the data presented in this review is drawn from systematic reviews. Since the inferences drawn in the review rely on these systematic reviews, it is important to assess the QC of these reviews such as clinical heterogeneity, statistical heterogeneity as well as models used for meta analysis. In some cases, the authors refer to systematic reviews from very few geographical regions of a country - e.g. Iran. How reliable are these studies? The reliability of the data presented is heavily dependent on the QC of these meta analyses. Therefore the authors need to ensure that they only present data from robust systematic reviews.

**Author Response: **Thank you for your comment. As suggested, we have assessed the articles (systematic reviews/meta-analysis) which were used in the manuscript. All studies included in this review were found to be reliable. Heterogeneity was assessed in each study and further analysis was performed accordingly. Sensitivity analysis and subgroup analysis were performed even if there was marginal heterogeneity. Quality assessment was performed for most studies included in systematic reviews and score was acceptable in each review. Regarding the systematic review and meta-analysis conducted among Iranian children from three geographical regions of the country, the study was found to be robust with a good quality score of 8 and no publication bias (as assessed via a funnel plot). So, overall, all systematic reviews are reliable in terms of heterogeneity and analysis. We have also added this statement to the Introduction section (Page 3). Our assessment has also been briefly compiled below (we had made a table but since we are not able to upload that via the journal portal we have copy-pasted the column information below):

**Study: **Avarvand et al., 2021

**Number of studies included: **7 studies

**I ^2^/Heterogeneity: **There was a significant heterogeneity among the studies (P<0.001; I2 = 98.26%).

**Mitigation:**

 1. Random effect model to mitigate heterogeneity impact

 2. Subgroup analysis e.g., for geographical regions and detection methods for prevalence

 3. Sensitivity analyses were performed by removing one study at a time to evaluate the impact of each study on the summary results and between-study heterogeneity

**Interpretation in terms of significant heterogenous (Yes/No): **This SLR included good quality papers and heterogeneity was assessed via I2 test (P<0.001; I2 = 98.26%) so random effect model was used along with subgroup and sensitivity analysis. Publication bias was also assessed by funnel plot, which was symmetric, showed no evidence of publication bias and confirmed by the results of Begg’s rank correlation (z = 0.30, P = 0.76) and Egger’s regression result (t = 0.32, P = 0.76).

**Study: **Weinberger et al., 2010

**Number of studies included: **9 studies

**I ^2^/Heterogeneity:**

 1. For Pneumonia risk estimation: All of the serotypes exhibited low levels of heterogeneity among studies based on the I2 values, with the exception of serotypes 9V and 23F, which exhibited moderate levels of heterogeneity.

 2. Foe meningitis risk estimation: Overall, substantial heterogeneity among studies was not there.

**Mitigation:**

 1. Random effect model and fixed effect model both were used to compare impact of heterogeneity

 2. Logistic regression

**Interpretation in terms of significant heterogenous (Yes/No): **No significant heterogeneity so considered

**Study: **Ballsells at al., 2018

**Number of studies included:**13 datasets (9 included data provided by collaborators and 4 from published studies)

**I ^2^/Heterogeneity: **Heterogeneity for each serotype included in the meta-analyses using the I2 where values of less than 25% indicate low heterogeneity, of 25% to 50% as moderate and above 50% as considerable heterogeneity.

 Heterogeneity was negligible to moderate for serotypes with a higher or lower invasive disease potential than 19A, except for 12F, 15A, 15BC. Sensitivity analyses did not influence the heterogeneity for these meta estimates.

**Mitigation: **Random effect model for meta-analysis was used as substantial heterogeneity in the included studies was anticipated.

 Sensitivity analyses were conducted to explore the effect of differences across datasets on overall meta-estimates by restricting analysis to datasets with the following characteristics: a) ≥70% PCV coverage, b) low prevalence of HIV, c) industrialized country settings, d) case counts from years subsequent to introduction of a higher valent PCV (10/13), e) implementation of PCV10 or PCV13

**Interpretation in terms of significant heterogenous (Yes/No): **Less heterogeneity so considered

**Study: **Chen et al., 2021

**Number of studies included: **26 studies

**I ^2^/Heterogeneity: **Heterogeneity evaluated using I2 statistics was interpreted as follows: I2= 0%, no heterogeneity; I2> 0% but < 25%, minimal heterogeneity; I2≥ 25% but < 50%, mild heterogeneity; I2≥ 50% but < 75%, moderate heterogeneity; and I2≥ 75%, strong heterogeneity.

**Mitigation: **For outcome of mortality:

 • Age >64 vs <65 years: I2 32.2%

 • State of septic shock at admission vs no septic shock: I2 0%

 • non-community-acquired infection vs community-acquired infection: I2 10.1%

 • Underlying chronic diseases: I2 72.9%

**Interpretation in terms of significant heterogenous (Yes/No): **Acceptable quality and heterogeneity of studies so considered

**Study: **Chamkhaleh et al., 2020

**Number of studies included: **8 studies

**I ^2^/Heterogeneity: **NA

**Mitigation: **NA

**Interpretation in terms of significant heterogenous (Yes/No): **Acceptable quality of studies so considered

**Study: **Johnson et al., 2010

**Number of studies included: **160 studies

**I ^2^/Heterogeneity: **Homogenous data: In Africa, ten serotypes had an ICC (Intraclass correlation) <0.2, thus the meta-estimates for these ten serotypes are particularly sensitive to the addition or removal of serotype data in this region. Asia and LAC also had a large number of serotypes with ICC<0.2 (six and five serotypes, respectively). By contrast, in Oceania, only serotype 14 had an ICC<0.2, indicating relative homogeneity in the reported serotype proportion data for this region. 

**Mitigation: **Marginal random-effects meta-analysis

**Interpretation in terms of significant heterogenous (Yes/No): **No heterogeneity with 160 studies data so considered

**Study: **Balsells et al., 2017

**Number of studies included: **68 studies

**I ^2^/Heterogeneity: **Datasets were heterogenous

**Mitigation: **Random effects model (DerSimonian-Laird method).  Heterogeneity was addressed by analyzing the most comparable data and case definitions. Analysis was stratified by PCV vaccine formulation and used data for children of the same age (<5 years) whenever possible. Sensitivity analysis was performed.

**Interpretation in terms of significant heterogenous (Yes/No): **Though data was heterogenous but was having good amount of data and analyzing comparable data only so considered.

 2. Section on pneumococcal serotypes - I suggest that the authors tabulate the pneumococcal serotypes associated with colonization vs. invasive disease along with the supporting references. 

**Author Response: **The authors would like to thank the reviewer for this suggestion. We have created Table 1 (Page 5) which provides the “Colonization or invasive potential of *S. pneumoniae* serotypes”. As per journal guidelines all tables have been shared in a separate document.

 3. Many times the authors list some serotypes as frequently associated with carriage and IPD. This does not make sense.  For e.g. page 6, under data from Iran, 19F is classified into both IPD and carrier populations.  Similarly, serotype 23F is listed under both carriage and IPD.

**Author Response: **Thank you for your comment. Both serotypes 19F and 23F cause invasive disease but are also common in carriage (Sandgren et al., 2004). This can be clearly visualized in the newly created tables 1 and 3 of the revised manuscript. The ability of pneumococcal serotypes to colonize or cause invasive disease may be influenced by both host-derived as well pathogen-derived factors. As per published literature, both serotypes 19F and 23F have a low invasive potential and are primarily found in carriers (Burgess et al., 2008). These serotypes are known to be associated with an impaired antibody response following invasive pneumococcal disease (Littorin et al., 2018; Soininen et al., 2001). Further, in addition to the capsular serotype, the clonal type may also determine the disease causative potential of pneumococcal serotypes as published by Sandgren et al. (2004) who reported that clones belonging to the same serotype showed different abilities to cause invasive disease.

 4. For the data on countries from EMR region, age-wise stratification of serotype prevalence and correlation with antibiotic resistance profile is needed.

**Author Response: **Thank you for your suggestion. We have incorporated Table 3 into the manuscript (Pages 9−11) which provides an “Age-wise stratification and antibiotic resistance among *S. pneumoniae* isolates in the Eastern Mediterranean Region”. As per journal guidelines all tables have been shared in a separate document.

 5. The authors need to refrain from claiming that they can draw inference for Iraq from neighboring countries since although regions are located geographically closer, serotype replacement and capsule switching are regulated by selection pressure unique to the population. Even in this review there are unique serotypes associated with Iraq such as NVT 33C that is not found elsewhere in the region. 

**Author Response: **The authors would like to thank the reviewer for this suggestion. We have removed this statement from the abstract and from the Introduction section (Page 3, paragraph 2) and modified the paragraph to ensure readability.  

# Reviewer report: Reviewer response for version 1

**DOI:** 10.5256/f1000research.145730.r174929

**Published:** 2023-06-15

**Authors:** Godfrey M. Bigogo

**Affiliations:** 1 Centre for Global Health Research, Kenya Medical Research Institute, Kisumu, Kenya

The authors sought to describe the Streptococcus pneumoniae serotype landscape in Iraq where there is inadequate surveillance for this pathogen. Published data from Iraq's neighbors is extensively used. Citations are properly done for all data used in the article. Two suggestions for the authors to consider:

1. I would strengthen the conclusion by stating that the serotypes in Iraq are likely to be similar to those circulating in the neighboring countries due to similarities including ecological conditions, cultural practices (like mass gatherings during worship). 
2. Is burden data available for any of Iraq's neighbors (Iran, Kuwait, Saudi Arabia etc.). If so, how does that compare with Iraq's IPD incidence of 0.62/100,000?

Minor issue:

 The opening statement in the Introduction could be rewritten as follows;

 "....for devastating morbidity and mortality among children below five years of age, adolescents, adults above 60 years of age, the immunocompromised population...".

Is the review written in accessible language?

Yes

Are all factual statements correct and adequately supported by citations?

Yes

Are the conclusions drawn appropriate in the context of the current research literature?

Partly

Is the topic of the review discussed comprehensively in the context of the current literature?

Yes

Reviewer Expertise:

Epidemiology of infectious diseases

I confirm that I have read this submission and believe that I have an appropriate level of expertise to confirm that it is of an acceptable scientific standard.

# Response

**Published:** 2023-12-19

**Authors:** Delan Ikram

**Affiliations:** Pfizer, Iraq

1. I would strengthen the conclusion by stating that the serotypes in Iraq are likely to be similar to those circulating in the neighboring countries due to similarities including ecological conditions, cultural practices (like mass gatherings during worship). 

**Author Response: **Thank you for your kind comment. This statement has been added to the conclusions section (Page 15): “Pneumococcal serotypes in Iraq are likely to be similar to those circulating in the neighboring countries due to similarities including ecological conditions and cultural practices like mass gatherings during worship. Additionally, based on a comment by the second reviewer we have also added the following statement: “However, serotype replacement and capsule switching are regulated by selection pressure unique to the population.”

 2. Is burden data available for any of Iraq's neighbors (Iran, Kuwait, Saudi Arabia etc.). If so, how does that compare with Iraq's IPD incidence of 0.62/100,000?

**Author Response: **The authors would like to thank the reviewer for this comment. As per the available literature, the prevalence of invasive *S. pneumoniae* infections was found to be low (2.5%) among children in Iran (Avarvand et al., 2021). This data is already available in the section on Iran (Page 12). In Saudi Arabia, the incidence of confirmed IPD cases was estimated to be 2.5−21.6 per 100,000 children (<5years) (Almazrou et al., 2016). This has now been added to the section on Saudi Arabia (Page 14) as: “A multicenter, prospective study conducted between June 2007 and January 2009 estimated the incidence of confirmed IPD cases to be 2.5−21.6 per 100,000 children <5 years.” Its reference has been added to the end of the reference list as per journal guidelines. Further we have also added a statement to Page 8: “However, inadequate surveillance as well as inconsistent reporting methods are associated with an underestimation of pneumococcal diseases burden in the Gulf countries (Mokaddas et al., 2018)”.

 3. Minor issue: The opening statement in the Introduction could be rewritten as follows; "....for devastating morbidity and mortality among children below five years of age, adolescents, adults above 60 years of age, the immunocompromised population...".

**Author Response: **Thank you for your suggestion. The opening statement in the Introduction (Page 2) has now been modified as follows: “ *Streptococcus pneumoniae*, the causative agent of pneumococcal disease, is responsible for devastating morbidity and mortality among children <5 years of age, adolescents, adults >60 years of age, the immunocompromised population, patients with chronic diseases, and smokers.”
